# New medical therapies on the horizon: oral octreotide

**DOI:** 10.1007/s11102-016-0785-3

**Published:** 2017-01-16

**Authors:** Nienke R. Biermasz

**Affiliations:** Division of Endocrinology, Department of Internal Medicine, Leiden University Medical Center, BOX 9600, 2300 RC Leiden, The Netherlands

**Keywords:** Pituitary, Octreotide, Acromegaly

## Abstract

Somatostatin analog treatment is first line medical treatment in patients with acromegaly. This drug is currently mainly administered by monthly depot preparations of octreotide and lanreotide. With the innovative transient permeability enhancer, a technology enabling the absorption of drug molecules via transient opening of the tight junctions of the gut epithelium, it is possible to achieve therapeutic octreotide levels after oral ingestion. The present review summarized the preclinical work and the recently reported phase I and III study on oral octreotide capsules in patients with acromegaly. Maintenance of control in 155 participating patients was achieved in 65% at the end of core period. Once controlled on oral octreotide, the response was maintained to the end of the extension phase in 85%. Side effects were comparable to currently available preparations. There was a profound suppression of growth hormone levels, and significant symptom reduction. Currently available parental somatostatin analogs are generally well tolerated and are able to achieve longstanding biochemical control in patients with somatostatin sensitive tumors. Potential advantages of an oral alternative is the lack injection-related side effects, but there will be an ongoing need for a very strict compliance with the 2 daily dose regimen and fasting around drug administrations. A second phase III study is currently being conducted. The potential place in the treatment of acromegaly is discussed.

## Introduction

Octreotide is an analog of the natural somatostatin with high binding affinity for the SSTR2 and moderate binding to SSTR5 receptor, but with longer half-life. Hypothalamic hormones growth-hormone releasing hormone GHRH, gut hormone Ghrelin (stimulating) and somatostatin (inhibiting) are key players in the regulation of GH synthesis, basal and pulsatile secretion in physiology. Octreotide was introduced for the treatment of acromegaly in the 80 s. Initially, only short acting subcutaneous preparation were available requiring a three-daily injection regimen but in later stage an intramuscular depot preparation using microspheres was introduced, facilitating the use of the drug. Somatostatin and its analogs, i.e. octreotide and lanreotide, strongly suppress GH release in healthy controls and in patients with acromegaly.

Somatostatin analogs are first line medical treatment of acromegaly [1]. The long-term efficacy and safety have been evaluated in landmark studies, open label studies and summarized in numerous reviews, with 40–60% of patients achieving normalization of predefined GH and IGF-I targets reflecting a generally accepted level of control [2]. A significant proportion of adenomas show a variable degree of adenoma shrinkage, and during long-term somatostatin analog treatment tumor growth is rarely observed. The safety profile of octreotide and other somatostatin analogs (SRLs) is fairly good, with mainly side effects that are a result of the selective activation of the receptors, that are also present in the GI system, i.e. bile stones, mild effects on glucose metabolism and gut motility, and furthermore local injection site related side effects.

The chance to achieve control is mainly dependent on the SSTR receptor status of the adenoma, and somewhat on the height of pretreatment GH levels. Other factors include young age, large tumors, and certain MRI and pathological characteristics (hyperintensity on T2 weighted imaging and sparse granularity) [3]. Usually there is a variation in SRLs responsiveness within a continuous spectrum, the vast majority being either good or partial responsive to SRLs. This is exemplified for example in studies evaluating single doses of octreotide, i.e. octreotide tests [4]. Therefore also trial design, i.e. which patients are included (preselection on octreotide sensitivity), and criteria that define remission, are important factors that influence differences in reported remission rates of individual studies [5]. There is only a small subset of adenomas that is truly SRL resistant, usually because SSTR2 expression is lacking or low. Pasireotide, an SRL with increased affinity for SSTR5 and other SSTRs may have additive values over octreotide or lanreotide in these situations.

### Oral octreotide

Orally ingested octreotide fails to achieve therapeutic drug levels following absorption in the jejunum, due to the intestinal barrier, only very low concentrations are measured. In early development of octreotide some, but too low and very variable, enteral absorption was reported and strategies to improve enteral absorption have not been successful up to now [6, 7]. The oral route has regained new interest with the introduction of the TPE technology, a transient permeability enhancer (TPE) consisting of among others medium-chain free fatty acids, sodium caprylate, inert excipients. Octreotide is encapsulated in an enteric coating, preventing breakdown before reaching the small intestine. The TPE is able to facilitate absorption of drug molecules and the combination with octreotide is the first proof of concept study. Due to transient opening of intestinal epithelial tight junctions drug molecules, as octreotide can traverse the tight junctions resulting in improved absorption, but absorption is dependent on molecular size. The current literature on this investigational drug from the first preclinical to the finished Phase III trial and a recent review on new therapeutic agents will be summarized in this review. The drug is not yet approved for use and a new Phase III study is currently being conducted (clinical trials.gov: MPOWERED, NCT02685709).

### Preclinical studies

Pharmacokinetic/ dynamic studies in rats and primates demonstrated a comparable pattern of octreotide drug levels between ingestion of oral octreotide capsule and sc octreotide injection [8]. Both modes of delivery show a rapid and sustained (complete) suppression of GH concentrations for several hours. Safety studies in cynomolgus monkeys showed no toxic effects on organs and a comparable profile of oral octreotide capsules and subcutaneous injection. The gastrointestinal mucosa and epithelium was not affected.

Using fluorescent tracers in a rat model the effect of TPE on tight junctions is visualized and these experiments illustrate the mechanism of action of TPE. There is a transient reorganisation of the cytoskeleton for 1–2 h, with normalization afterwards. The molecular size in relation to absorption was assessed with dextran at several molecular weights, with best absorption present with smallest dextrans (4 kDa). Larger dextrans (40–70 kDa) were only minimally absorbed, limiting the risk of internalization of intestinal pathogens and immunoglobulins. The short action and selectivity with respect to size are two important aspects to consider with respect to safety [8].

### Phase I study

Single dose studies were conducted in 75 healthy volunteers using doses of 3, 10, 20 mg oral octreotide and 100 µg sc injection. There was a dose–response effect of the 3 different doses on plasma octreotide levels with PK parameters proportional to the oral dose administered. Levels were detectable after 30 min of ingestion. Capsules of 20 mg and sc injection of 100 µg provide comparable plasma concentrations. After a 20 mg capsule levels peaked at 2.7 h, somewhat later than sc injection, with therapeutic levels upto 8 h. It was concluded that a two daily dose scheme would suffice. There is a significant interference with food intake as well as with proton pump inhibitors resulting in decreased bioavailability. Oral octreotide was able to suppress low basal GH secretion in healthy controls and strongly inhibited the peak GH elicited by GHRH-arginine stimulation from 50 to 10 ng/ml as well as suppressed the GH (AUC). Safety profile was comparable except for the lack of injection site related discomfort [9].

### Phase III study

In a baseline-controlled open-label, multicentre Phase III study 155 patients were enrolled who were treated with stable doses (>3 m) of injectable SRL treatment and had sensitivity to octreotide as evidenced by full or partial biochemical control (IGF < 1.3 ULN, GH < 2, 5 ug/L) [10]. The main goal of the trial was to show maintenance of control of GH and IGF-I baseline levels during treatment with oral octreotide capsule treatment. Four weeks after a last regular injection, patients were switched to oral octreotide 40 mg (20 mg twice daily, >1 h before and >2 h after a meal). In the titration phase, patients were titrated up to 80 mg daily if necessary to maintain GH and IGF-I levels. Once stable, patients entered the maintenance phase with fixed doses continued to the end of the core study at 7 months. The core study period was followed by a voluntary extension phase of 6 months, resulting in a period of 13 months of stable drug treatment. 86% percent of patients elected to continue with this extension phase. The modified intention to treat group (cohort with at least one efficacy measure on capsules, mITT) showed maintenance of biochemical response (i.e. IGF-I < 1.3 ULN and GH < 2.5 µg/L) in 65% of patients at end of core treatment and 62% at end of extension period. 85% of patients that could enter the fixed dose phase sustained response upto 13 months. Both in the mITT and the fixed dose populations, in the presence of unchanged IGF-I levels GH levels showed a profound suppression during oral octreotide, throughout the entire study period.

Clinical control of acromegaly related symptoms (headache, asthenia, perspiration, swelling of extremities and joint pain) improved during the trial. Despite biochemical partial or full biochemical control baseline symptoms were present in 81% of patients on regular SRL injectables. By the end of the trial the severity of symptoms had decreased from baseline: 26% maintained, 54% improved the symptom severity score. Also the proportion of subjects with at least one, two, or three acromegaly symptoms decreased from 79, 63, and 45%, respectively, at baseline on injectables to 68, 48, and 31% at the end of study during treatment with oral octreotide capsules. There is only partial correlation with IGF-I levels or IGF-I control and symptoms [11].

Adverse events were compatible with the known side effect profile of octreotide or related to the disease. Most common side effects were gastrointestinal (nausea and diarrhoea), neurological (headache) and musculoskeletal (arthralgia) and observed in the earlier treatment period, resolving with continued treatment. Ten patients discontinued because of GI side effects, 21 patients sustained a serious adverse event, and two patients died during the trial supposedly unrelated to study drug.

Detailed analysis of factors influencing (non)-responsiveness on octreotide capsules in this cohort of patients previously controlled on injectables was not included in the phase III study paper, however, uptonow some additional exploratory analysis have been performed and these have been published as meeting abstracts.

In general, octreotide sensitivity is mainly determined by somatostatin receptor subtype status of the tumor, and achievement of defined biochemical control criteria are dependent on this status, and also the disease activity (height of GH and IGF-I levels) and drug levels. There are as yet no clues that these factors are different for oral octreotide or injectables.

Mode of delivery specific issues for the oral route related to non-responsiveness are likewise, compliance and insufficient drug absorption. In the trial, these issues were vigorously monitored and instructions were given to prevent interaction with food and drugs. In the trial a subset of patients had a PK analysis, confirming that a two daily dosing regimen provides therapeutic drug levels. It is of note that GH was evaluated just after oral octreotide capsule intake, and GH levels were firmly suppressed and so drug absorption was adequate. Firm GH suppression, suggestive for adequate octreotide absorption, was also present in non-responders, which was based on IGF-I criterion in the majority of non-responders. The duration of GH suppression following ingestion was not evaluated.

Theoretically, trial specific issues to explain non-responsiveness to some extent could be the trial design and the single time point and timing of GH curve used to classify patients. These factors are not unique to this specific trial. The baseline controlled trial without withdrawal period to wean injectables did not allow further dose increase once a patient had entered the maintenance phase. Since the carry over effect of injectables may last for many weeks, it cannot be fully excluded that some of the non-responders on the low and medium oral octreotide doses would have been responders at higher doses. Hormone levels fluctuate from time to time, and especially in patients with levels just around the cut-off level evaluation by single time point may influence the classification of responder and (non) responder both at entry and at end of trial.

Factors influencing the response to oral octreotide was assessed in an exploratory analysis, presented at the meeting of the Endocrine Society in 2015. Baseline biochemical control on injectable SRLs (IGF-I < 1 and GH < 2.5) and use of low to mid dose SRL injectables predicted subsequent favorable response to oral octreotide capsules, i.e. 84.5% (49 of 58 subjects) [12]. The dose of oral octreotide required was related to baseline SRL dose. It is of note that some (24%) of patients that were classified at non-responder at baseline on injectables were responders at the end of the study on oral octreotide.

The GH criteria was met in the vast majority of patients on oral octreotide and non-responsive was mainly based on a failure to meet the IGF-I criterion. Although discrepancies in GH and IGF-I have been also described with injectables, this appears to be somewhat more pronounced during oral octreotide. Potential explanations are variable (duration of) suppression of GH pulsatility, particular during troughs of drug concentrations of both capsules (end of daily dose) and injections (and of monthly injection) and direct action of octreotide on the liver IGF-I production. Further experience with oral capsules need to clarify whether this differential effects of GH and IGF-I suppression are confirmed and whether these factors are of clinical relevance.

### Potential place of oral octreotide in the treatment of acromegaly

At present oral octreotide capsules are an investigational drug, therefore the place in the treatment algorithm is hypothetical. Any differences between oral versus long-acting injectable SRLs are related to the administration route, frequency and the different pharmacokinetic profile, and both options may have advantages and disadvantages and will have individual preferences. However, both act on the same SSTR receptor subtypes and since sensitivity to the drug will be mainly dependent on the receptor status and post receptor machinery and will therefore be more or less comparable, once therapeutic drug levels are achieved [3]. According to the results of the phase III study the majority of patients controlled on injectables—except for the small subset that experienced unacceptable side effects (10%) and patients that loose control possibly due to inadequate drug levels, low absorption, compliance etc. (35%)—can be treated with either injectables or oral capsules and advantages and preferences would need to be carefully weighed to the personal situation, once both drug delivery methods would be available.

In a PRO study the patient perspective on use of injectables was explored in the chronically treated cohort of acromegaly patients [13]. Many patients have active symptoms while on injectables and some reported aggravation of symptomatology towards the end of the injection interval, so called wear off phenomenon. Burden due to injection related symptoms were reported, although the majority of patients is satisfied with current treatment with injectables. Avoiding injections and better symptom control are two reported unmet needs.

The two daily regimen of capsules requires ongoing and strict compliance of patients, which needs ongoing effort, and needs to be monitored probably more intensively than treatment with injectables to prevent episodes of uncontrolled acromegaly. On the other hand it will provide patients tools to prevent the experienced instability in control during monthly injections (wear off effect), and may be easier to titrate and withdraw than the very long-acting injections requiring months to achieve a plateau and to withdraw.

The profound suppression of GH observed in oral octreotide that may indicate a different dynamic of the GHIGF-I axis is a very interesting observation, requiring further study to understand consequences at tissue level and receptor status. It could be related to the observed improvement in symptoms but need further investigations to draw conclusions. There are no published data on the effect of octreotide capsules on tumor shrinkage.

The improved symptom status during the trial needs to be interpreted with caution, because it was an open label study and close monitoring of the patients during the trial may bias this observation. The correlation between symptoms and biochemical status is not straightforward, but symptoms are a very relevant clinical endpoint, although difficult to measure because of the fact that symptoms may be only partly reversible and are likely multifactorial.

It is important to note that also any chosen biochemical endpoints of any acromegaly trials and trial methodology impact efficacy rates, because patients classified as controlled may be classified as uncontrolled at a sequential time point during the same treatment [2, 14]. This is for example illustrated by the difference in biochemical response rate between screening and baseline in the phase III oral octreotide study [10]. This is also illustrated by the variation in levels throughout the trial. In a posthoc analysis presented at the European Congress of Endocrinology, Munchen, 2016, the time weighted average response was proposed as a more sophisticated way to evaluate response. It is calculated by using all available IGF-I and mean GH measurements of the core treatment period and by dividing the area under the curve by the total amount of time under observation. For example, in the mITT population of the phase III trial response rate was 64.9% (98 out of 151 patients) based on the single time point at the end of core treatment, when all available GH and IGF-I levels during core treatment were used for evaluation the response rate was 71.5%. Sixteen percent of patients were classified differently: 17 were responders using the time weighted average of all measurements but not when the last timepoint was evaluated and 7 were responder according to the last timepoint but not when evaluated with a time weighted average evaluation. Comparable figures were found when evaluating the fixed dose population. It was concluded that a time weighted average of different sequential values may be a more sophisticated measure to reflect chronic control in contrast to a single time point when taking into account the known variability of GH and IGF-I levels [15].

Systematic evaluation of clinical and (average) biochemical values in clinical practice are also needed to choose and monitor best individual treatment options. Recent tools, i.e. SAGIT, try to help physicians to optimally score the several aspects important for care in acromegaly [16].

It is likely that the patient perspective and patient voice will gain more and more attention in the chronic care of acromegaly as is in other chronic (endocrine) diseases and in view of an individualized approach in management the possibility of an effective oral treatment in acromegaly will be welcomed as an additional option for a yet unknown subset of patients [17].
